# Immunotherapy of ovarian cancer with a monoclonal antibody specific for the extracellular domain of anti-Müllerian hormone receptor II

**DOI:** 10.18632/oncotarget.27585

**Published:** 2020-05-19

**Authors:** Suparna Mazumder, Valerie Swank, Anton A. Komar, Justin M. Johnson, Vincent K. Tuohy

**Affiliations:** ^1^ Department of Inflammation and Immunity, Lerner Research Institute, Cleveland Clinic, Cleveland, OH, USA; ^2^ Department of Molecular Medicine, Cleveland Clinic Lerner College of Medicine of Case Western Reserve University, Cleveland, OH, USA; ^3^ Case Comprehensive Cancer Center, Cleveland, OH, USA; ^4^ Department of Biological, Geological and Environmental Sciences, Cleveland State University, Cleveland, OH, USA; ^5^ Center for Gene Regulation in Health and Disease, Cleveland State University, Cleveland, OH, USA

**Keywords:** monoclonal antibody, anti-Müllerian hormone receptor type II, immunotherapy, ovarian cancer, 4D12G1

## Abstract

Epithelial ovarian carcinoma (EOC) is the most prevalent and lethal form of ovarian cancer. The low five-year overall survival after EOC diagnosis indicates an urgent need for more effective ways to control this disease. Anti-Müllerian hormone receptor 2 (AMHR2) is an ovarian protein overexpressed in the majority of human EOCs. We have previously found that vaccination against the ovarian-specific extracellular domain of AMHR2 (AMHR2-ED) significantly inhibits growth of murine EOCs through an IgG-mediated mechanism that agonizes receptor signaling of a Bax/caspase-3 dependent proapoptotic cascade. To determine if a single monoclonal antibody (mAb) could inhibit growth of human EOC, we generated a panel of mAbs specific for recombinant human AMHR2-ED and characterized a candidate mAb for humanization and use in clinical trials. We found that our candidate 4D12G1 mAb is an IgG_1_ that shows high affinity antigen-specific binding to the 7-mer ^20^KTLGELL^26^ sequence of AMHR2-ED that facilitates induction of programmed cell death in EOC cells. Most importantly, the 4D12G1 mAb significantly inhibits growth of primary human EOCs in patient-derived xenografts (PDXs) by inducing direct apoptosis of EOC tumors. Our results support the view that a humanized 4D12G1 mAb may be a much needed and effective reagent for passive immunotherapy of human EOC.

## INTRODUCTION

Epithelial ovarian carcinoma (EOC) is the most lethal of all gynecologic malignancies with postmenopausal women accounting for more than 75% of all cases [1, 2]. An array of non-definitive symptoms associated with EOC onset and the lack of effective biomarkers for early detection often results in late diagnoses at advanced diseased stages resulting in high rates of disease recurrence and poor prognoses following current standard of care that typically includes platinum-based drugs [1–3]. Although often effective initially, toxicity and/or resistance to platinum-based drugs is common and frequently leads to alternative treatments with a variety of different therapeutics that have historically provided variable and short-lived results [4, 5]. More recently, substantial success in controlling EOC progression has been achieved following treatment with inhibitors of poly (adenosine diphosphate-ribose) polymerase (PARP), a family of proteins involved in DNA repair [6]. However, resistance to PARP inhibitors has subsequently been described involving multiple mechanisms of action [7]. Thus, there remains a great need for more effective ways to control this disease. To this end, we and others have shown that anti-Müllerian hormone receptor type II (AMHR2) is expressed in 90% of primary EOCs, 78% of borderline malignancies, 77–86% of non-EOC ovarian tumors, and 56% of malignant ascites from grades III-IV ovarian cancers [8–11]. As such AMHR2 may serve as an effective target for immune control of primary and more advanced forms of EOC.

AMHR2 is a serine/threonine kinase receptor homologous to type II receptors of the transforming growth factor-beta (TGFβ) superfamily [12]. Anti-Müllerian hormone (AMH) is the cognate ligand of AMHR2, and binding of AMH to the extracellular domain of AMHR2 (AMHR2-ED) signals cell cycle arrest and programmed cell death resulting in regression of the Müllerian ducts during male development and regulation of oocyte development, and control of ovarian reserve and fertility in adult females [13, 14]. In adult women, the longest AMHR2 transcript codes for a 573 amino acid protein expressed exclusively in the ovary and consisting of the ovarian-specific 127 amino acid AMHR2-ED ligand binding domain, along with a 26 amino acid transmembrane domain, and a 403 amino acid cytoplasmic kinase domain (AMHR2-CD) both of which show extra-ovarian expression [15].

We have previously reported that vaccination against either the cytoplasmic domain of AMHR2 (AMHR2-CD) or AMHR2-ED significantly inhibits growth of autochthonous and transplantable murine EOC tumors, and that this EOC growth inhibition is accompanied by significantly enhanced overall survival [11, 16]. However, our attention has focused on the significant EOC tumor immunity generated following vaccination with AMHR2-ED because AMHR2-ED expression in normal human tissues is confined to the premenopausal ovary and its expression is *‘retired’* with age and is at non-autoimmunogenic levels in the human postmenopausal ovary. Thus, immunity directed against AMHR2-ED would preclude the development of extra-ovarian autoimmune complications in premenopausal women and would preclude all autoimmune complications in postmenopausal women. We found that AMHR2-ED vaccination inhibits the growth of EOC through the helper function of CD4 + T cells that facilitates B cells to produce AMHR2-ED specific IgG. The IgG agonizes a Bax/caspase-3 dependent proapoptotic signaling cascade known to inhibit tumor growth [16, 17].

Since we found that safe and effective immunity against ovarian tumors can be generated by AMHR2-ED-specific IgG, we generated a panel of monoclonal antibodies (mAbs) specific for recombinant human (rh) AMHR2-ED and characterized their binding and functional features to select a candidate mAb for use in human clinical trials focused on treatment of women with EOC. We found that the mAb clone 4D12G1 is an IgG_1_ that has several desirable features for humanization and use as our candidate mAb. These features include high affinity antigen-specific binding to AMHR2-ED in EOC cells and tissues and the ability to kill human EOC cells through 3 distinct immune mechanisms. Importantly, the 4D12G1 mAb is capable of significantly inhibiting growth of primary human EOCs in patient-derived xenografts (PDX). Collectively, our results indicate that the 4D12G1 mAb may be effective as a therapeutic reagent against human EOC.

## RESULTS

### Generation of mAbs specific for rhAMHR2-ED

**Figure 1:**
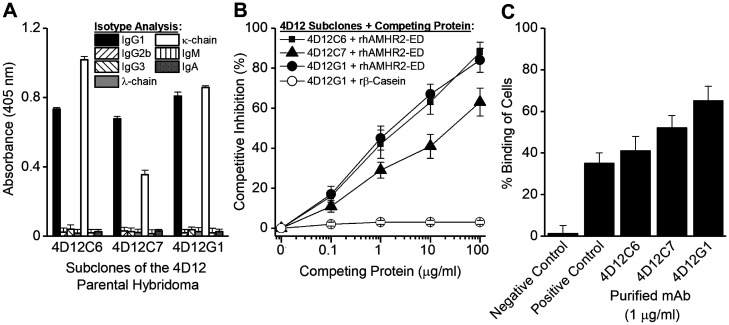
Generation of mAbs specific for rhAMHR2-ED. A panel of approximately 300 hybridoma supernatants were generated and screened for specificity against rhAMHR2-ED and the 4D12 parental hybridoma was selected for subcloning by limiting dilution. (**A**) Subcloning produced three sub-clones, 4D12C6, 4D12C7, and 4D12G1 each of which expressed the IgG_1_/κ-chain isotype and showed antigen specificity (**B**) by competitive ELISA and (**C**) by flow cytometry binding to OVCAR8 cells. For flow cytometry, positive control staining of OVCAR8 cells was performed using a commercially available anti-AMHR2-ED mAb (Abcam), whereas IgG1 isotype antibodies with irrelevant specificities were used as negative controls. In all cases, error bars indicate ± SD and the results shown are representative of three experiments yielding similar results.

Approximately 300 hybridoma supernatants were screened by ELISA for specificity against rhAMHR2-ED using recombinant mouse β-casein as the specificity control. Twelve hybridomas showed high-titer AMHR2-ED-specific responses. Based on flow cytometry binding of OVCAR8 cells (data not shown), the 4D12 parental hybridoma was selected for subcloning by limiting dilution. The subcloning of 4D12 produced the three sub-clones, 4D12C6, 4D12C7, and 4D12G1 each of which expressed the IgG_1_/κ-chain isotype (Figure 1A) and showed antigen-specificity in competitive ELISA (Figure 1B) and in flow cytometry binding to OVCAR8 cells (Figure 1C). The 4D12G1 mAb appeared to be superior in these initial binding studies and was examined further.

### The 4D12G1 mAb recognizes AMHR2 in human EOC

**Figure 2:**
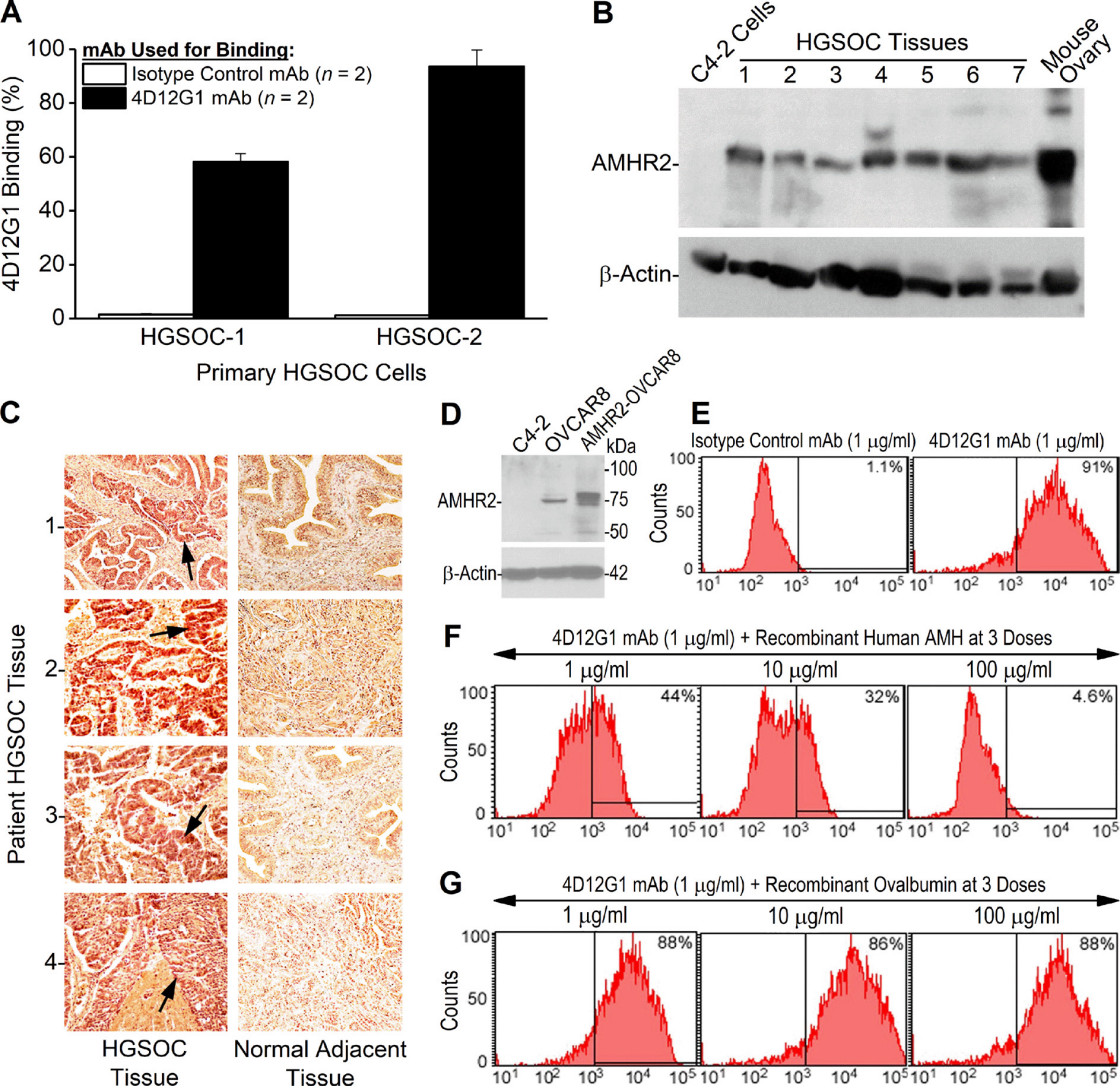
The 4D12G1 mAb recognizes AMHR2-ED in human EOC and competes with AMH for b inding to AMHR2-ED. (**A**) Flow cytometry analysis showing that the 4D12G1 mAb binds to the majority of cells generated from two primary HGSOC tissues examined. Error bars indicate ± SD. (**B**) The 4D12G1 mAb was used in Western blots of seven different HGSOC tissue lysates (25 μg protein/lane) with a positive control lysate generated from a young C57BL/6 ovary and a negative control lysate generated from C4-2 human prostate cancer cells. Immunostaining with a β-actin antibody was used to confirm normalized lysate loading. The Western blots shown are representative of three experiments that provided similar results. (**C**) The 4D12G1 mAb was used in immunohistochemical staining (20 ×) of tissue sections from four HGSOC patients (left column) and their normal adjacent fallopian tube tissues (right column). Arrows indicate staining of the tumor parenchyma. The stromal areas of the EOC tumors were not immunostained nor were all areas of the normal adjacent fallopian tube tissues. All experiments were performed three times yielding similar results. (**D**) Western blot analysis of lysates from OVCAR8 cells and AMHR2-OVCAR8 cells with lysates from C4-2 prostate cancer cells used as controls and immunostaining with a β-actin antibody was used to confirm normalized lysate loading. Flow cytometry analysis showed that: (**E**) the 4D12G1 mAb binds to 91% of AMHR2-OVCAR8 cells; (**F**) the AMH cognate ligand for AMHR2-ED effectively competes in a dose-dependent manner with the 4D12G1 mAb for binding to AMHR2-OVCAR8 cells; and (**G**) recombinant ovalbumin failed to compete with the 4D12G1 mAb for binding to AMHR2-OVCAR8 cells. Data are representative of three independent experiments yielding similar results.

The 4D12G1 mAb was used to detect AMHR2 in primary cultures of human EOC cells derived from fresh HGSOC tissues. Flow cytometry analysis showed that 4D12G1 detected AMHR2 in 58.2% of the primary HGSOC-1 cells and in 93.7% of the primary HGSOC-2 cells with isotype control mAb binding to only 1.5% and 1.1% of cultured cells, respectively (Figure 2A). By Western blot analysis, the 4D12G1 mAb immunostained seven different primary HGSOC tissue lysates as well as a positive control lysate derived from an ovary taken from a normal young C57BL/6 female mouse, thereby indicating the cross-reactive features of the 4D12G1 mAb in recognizing both human and mouse AMHR2-ED (Figure 2B). The 4D12G1 mAb did not immunostain a negative control lysate derived from the C4-2 human prostate cancer cell line. Immunostaining of β-actin was used to confirm normalized lysate loading. The 4D12G1 mAb was used in immunohistochemical staining of 13 primary HGSOC tissues from women with a mean age 60.2 years, range 38–76 years (Table 1) and in the normal fallopian tube tissues adjacent to the EOC tumors**.** The results obtained from patients 1–4 (mean age 64 years, range 59–70 years) indicate staining of the tumor parenchymas (Figure 2C, left column, arrows) with no staining of the stromal areas and no staining of any areas of the corresponding normal adjacent fallopian tube tissues (Figure 2C, right column). It is important to note that the four normal adjacent fallopian tube tissues that did not stain with the 4D12G1 mAb were from 59–70 year-old postmenopausal women, and therefore would not be expected to express AMHR2-ED, a protein domain ‘retired’ from expression in postmenopausal ovaries [16].

**Table 1:** Details of EOC patients and their examined tumors

HGSOC sample	Diagnosis^1^	Age^2^	Disease stage	Immunohistochemical intensity of AMHR2 expression
1	HGSOC	61	Primary	++
2	HGSOC	59	Primary	+++
3	HGSOC	66	Primary	+++
4	HGSOC	70	Primary	++
5	HGSOC	58	Primary	+++
6	HGSOC	38	Primary	+++
7	HGSOC	43	Primary	+++
8	HGSOC	76	Primary	+++
9	HGSOC	65	Primary	+++
10	HGSOC	65	Primary	+++
11	HGSOC	55	Primary	++
12	HGSOC	59	Primary	+++
13	HGSOC	68	Primary	++

^1^All examined tissues came from patients diagnosed with high grade serous ovarian carcinoma (HGSOC).

^2^Mean age of EOC patients = 60.2 years (range, 38–76).

### The 4D12G1 mAb competes with AMH for binding to AMHR2

Unlike primary human HGSOC tissues, human EOC cell lines typically show low expression levels of AMHR2. Thus, we transfected OVCAR8 cells with the full sequence of human AMHR2 to have a useful reagent for further analysis of the features of the 4D12G1 mAb. Western blot analysis of lysates from the stably transfected AMHR2-OVCAR8 cell line showed high level detection of AMHR2 protein compared to parental OVCAR8 cells with no detectable AMHR2 in lysates from the human prostate cancer cell line, C4-2 (Figure 2D). Flow cytometry analysis showed that the 4D12G1 mAb binds to 91% of AMHR2-OVCAR8 cells (Figure 2E), and competitive binding studies showed that the cognate recombinant AMH ligand for AMHR2 was able to compete effectively in a dose-dependent manner with the 4D12G1 mAb for binding to AMHR2-OVCAR8 cells (Figure 2F). This binding by the 4D12G1 mAb to the AMHR2-OVCAR8 cells was unaffected by dose-response treatment with recombinant ovalbumin thereby indicating the specificity of AMH for competing with the binding of the 4D12G1 mAb (Figure 2G).

### Identification of the AMHR2-ED binding site for the 4D12G1 mAb

**Figure 3:**
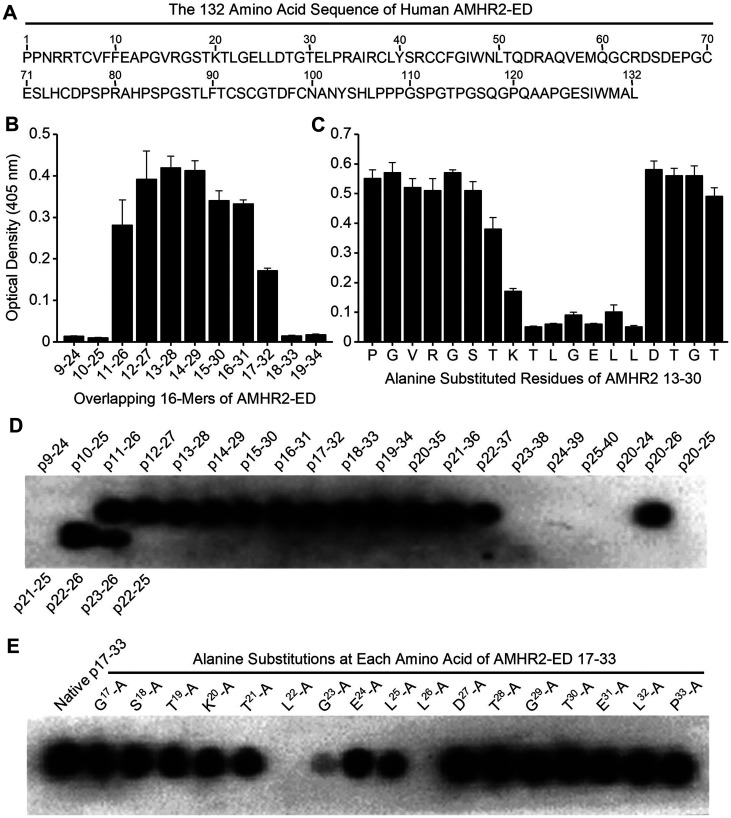
Identification of the AMHR2-ED binding site for the 4D12G1 mAb. (**A**) The entire 132 amino acid sequence of human AMHR2-ED. (**B**) An overlapping series of 16-mer peptides spanning the entire sequence of human AMHR2-ED with one amino acid shifts were plated for direct ELISA testing using the 4D12G1 mAb as the primary antibody. The 4D12G1 mAb recognized residues AMHR2-ED 11–32. (**C**) Overlapping peptides spanning AMHR2-ED 13–30 were synthesized with alanine substitutions at each N-terminal residue or with glycine substitutions for any native N-terminal alanine residues. Competitive ELISA results showed that alanine substitutions at residues spanning AMHR2-ED 20-26 (^20^KTLGELL^26^) decreased binding of the 4D12G1 mAb to AMHR2-ED. (**D**) SPOT peptide arrays using 4-16-mer peptides spanning AMHR2-ED 9-40 were immobilized on cellulose membranes, treated with the 4D12G1 mAb, and the bound antibody was detected by chemiluminescence. The results showed that the AMHR2-ED 22–26 5-mer sequence (^22^LGELL^26^) represents the minimal sequence for binding of the 4D12G1 mAb. (**E**) SPOT peptide arrays were made using membrane bound 17-mer peptides spanning the AMHR2-ED 17–33 domain and containing alanine substitutions at each sequential amino acid. Alanine replacement of Leu^22^, Gly^23^, and Leu^26^ completely abolished binding by the 4D12G1 mAb. All error bars indicate ±SD, and all experiments are representative of three experiments yielding similar data.

**Figure 4:**
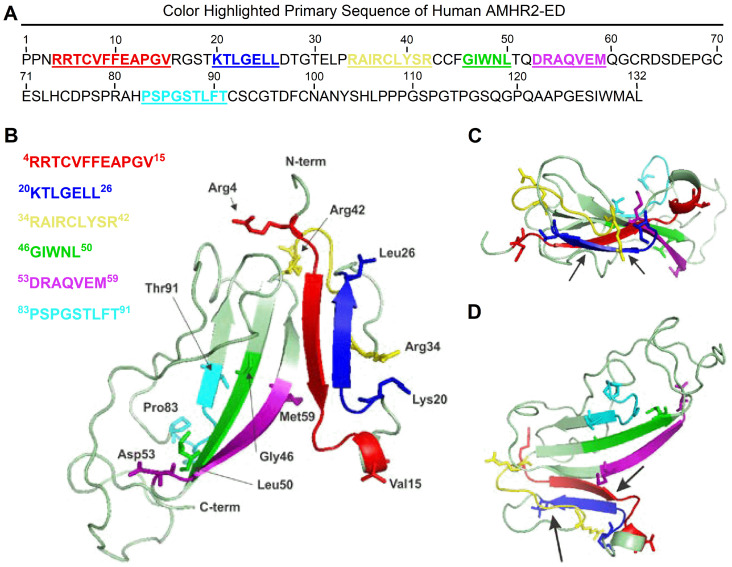
Molecular modeling of human AMHR2-ED. Proposed binding sites for the cognate AMH ligand include the key natural AMH binding site I, ^4^RRTCVFFEAPGV^15^ represented in red in (**A**) the primary amino acid sequence and as a red beta sheet in (**B**) the ribbon model juxtaposed but antiparallel to the ^20^KTLGELL^26^ binding site of the 4D12G1 mAb depicted in blue in both the primary sequence and as a beta sheet in the ribbon model. Another strong AMH binding site II, ^34^RAIRCLYSR^42^ is represented by a long yellow loop also adjacent to the ^20^KTLGELL^26^ binding site of the 4D12G1 mAb. In contrast, the ^53^DRAQVEM^59^ binding site of the 12G4 mAb and its 3C23K humanized and glyco-engineered variant (a. k. a. GM102) is closer to the weaker tertiary and quaternary AMH binding sites ^46^GIWNL^50^ and ^83^PSPGSTLFT^91^, respectively. Arrows point to the juxtaposition of the blue ^20^KTLGELL^26^ binding site of the 4D12G1 mAb with the red AMH I binding site, ^4^RRTCVFFEAPGV^15^ and the yellow AMH binding site II ^34^RAIRCLYSR^42^ in (**C**) a top view of the ribbon model as well as in (**D**) a 90° rotational view.

An overlapping series of 16-mer peptides spanning the entire 132 amino acid sequence of human AMHR2-ED (Figure 3A) with moving single amino acid shifts were plated for direct ELISA testing using the 4D12G1 mAb as primary antibody. The ELISA binding results showed that the 4D12G1 mAb recognized peptides spanning residues 11-32 ^11^EAPGVRGSTKTLGELLDTGTEL^32^ of AMHR2-ED (Figure 3B). To determine the critical amino acids recognized by the 4D12G1 mAb, an overlapping series of peptides spanning the immunoreactive sequence comprising AMHR2-ED 13–30 were synthesized with alanine substitutions at each N-terminal residue or with glycine substitutions for any native N-terminal alanine. These peptides were used in competitive ELISAs to determine which residues inhibit binding of the 4D12G1 mAb with solid phase rhAMHR2-ED. The results showed that alanine substitutions spanning AMHR2-ED 20–26 ^20^KTLGELL^26^ dramatically decreased binding of the 4D12G1 mAb to AMHR2-ED (Figure 3C). To determine the minimal AMHR2-ED sequence involved in the binding of the 4D12G1 mAb, SPOT peptide arrays were generated [18] spanning the immunoreactive region of AMHR2-ED. Peptides ranging from 4-16-mers in length were immobilized on cellulose membranes and treated with the 4D12G1 mAb. Bound antibody was detected by chemiluminescence. The results showed that AMHR2-ED 22–26 ^22^LGELL^26^ represents the minimal sequence needed for binding of the 4D12G1 mAb (Figure 4D). Additional binding details were obtained using SPOT arrays with membrane bound 17-mer peptides spanning the immunoreactive region and containing alanine substitutions at each N-terminal residue. The results showed that alanine replacement of Leu^22^, Gly^23^, and Leu^26^ residues of the ^20^KTLGELL^26^ sequence completely abolished binding by the 4D12G1 mAb (Figure 4E). Thus, L^22^, G^23^, and L^26^ represent essential residues for binding of the 4D12G1 mAb. Finally, we used surface plasmon resonance (SPR) to measure the equilibrium dissociation constant (K_**D**_) between the 4D12G1 mAb and AMHR2-ED at 119 pM (data not shown).

### Molecular modeling of human AMHR2-ED

To image the binding site region of the 4D12G1 mAb in the context of the entire AMHR2-ED protein, molecular modeling was performed using Phyre2 [19] and 3DLigandSite [20] tools and the highest confidence model was visualized using the PyMOL Molecular Graphics System, version 1.5.0.5 [20]. Proposed binding sites for the AMH cognate ligand include the key natural AMH I binding site spanning AMHR2-ED 4–15 ^4^RRTCVFFEAPGV^15^ represented in red in the primary amino acid sequence (Figure 4A) and as a red beta sheet in the ribbon model juxtaposed but antiparallel to the ^20^KTLGELL^26^ binding site of the 4D12G1 mAb depicted in blue in both the primary sequence (Figure 4A) and as a beta sheet in the ribbon model (Figure 4B). Another strong AMH II binding site spanning AMHR2-ED 34–42 ^34^RAIRCLYSR^42^ is represented by a long yellow loop also adjacent to the ^20^KTLGELL^26^ binding site of the 4D12G1 mAb (Figure 4B). The weak AMH III binding site spanning AMHR2-ED 46-50 ^46^GIWNL^50^ in green and an even weaker AMH IV binding site spanning AMHR2-ED 83–91 ^83^PSPGSTLFT^91^ in cyan are located quite distant from the ^20^KTLGELL^26^ binding site of the 4D12G1 mAb (Figure 4B). The juxtaposition of the blue ^20^KTLGELL^26^ binding site of the 4D12G1 mAb with the red AMH I binding site ^4^RRTCVFFEAPGV^15^ and the yellow AMH II binding site ^34^RAIRCLYSR^42^ is also evident in a top view of the ribbon model (Figure 4C) as well as in a 90° rotational view (Figure 4D). Thus, the AMHR2-ED binding site recognized by the 4D12G1 mAb is in close proximity to the key primary and secondary AMH binding sites, a feature that likely accounts for the ability of AMH to compete with the 4D12G1 mAb for binding to AMHR2-ED (Figure 2F). This view is supported by the fact that another characterized mAb against AMHR2-ED, the 12G4 mAb, is unable to compete with AMH for AMHR2-ED receptor binding likely because it recognizes the ^53^DRAQVEM^59^ sequence of AMHR2-ED (purple in Figure 4A through D) far from the strong AMH I and AMH II binding sites and much closer to the AMH III and AMH IV binding sites associated with weak binding of AMH to AMHR2-ED [21].

### The 4D12G1 mAb induces programmed cell death of EOC cells

**Figure 5:**
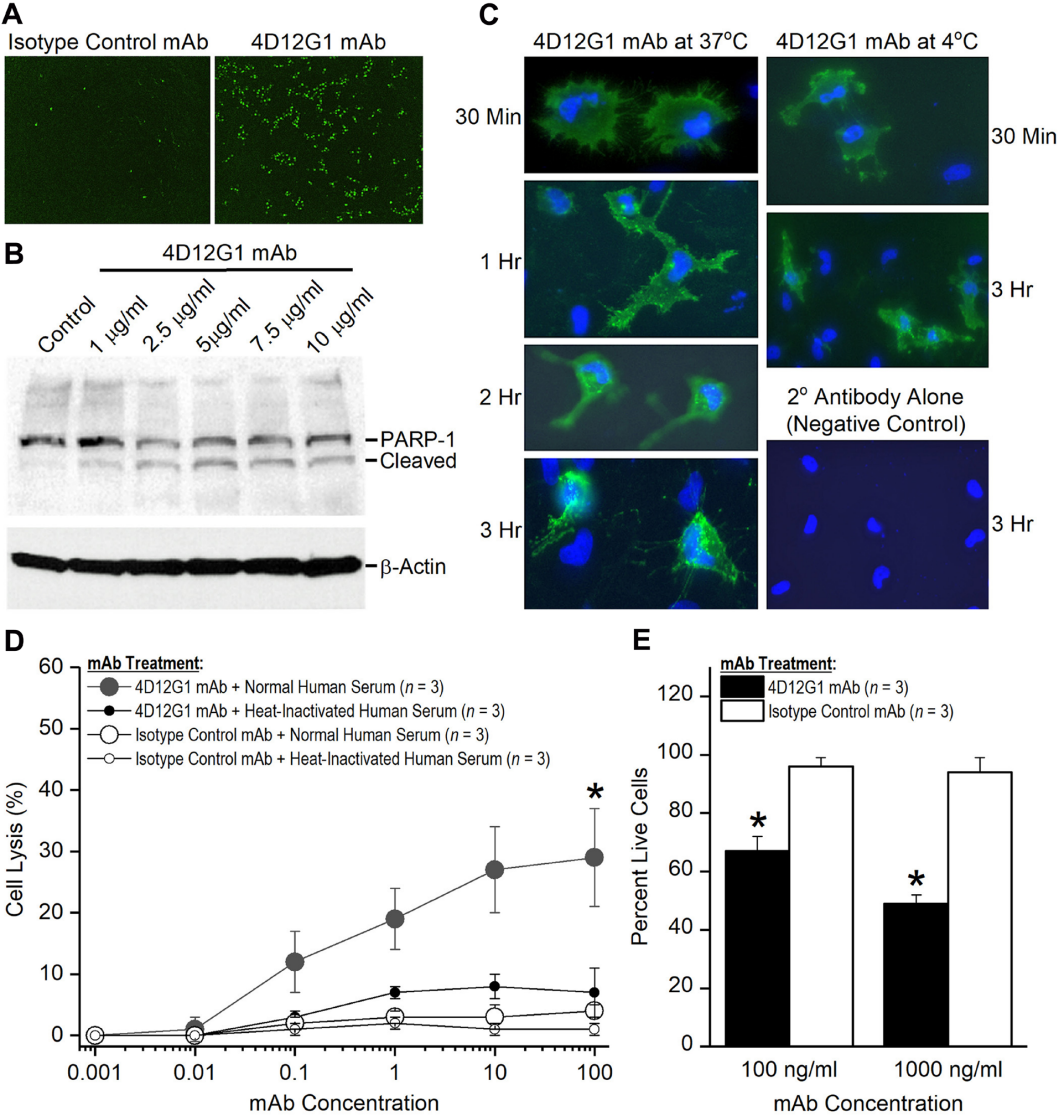
The 4D12G1 mAb kills EOC cells by inducing apoptosis, CDC, and ADCP. (**A**) AMHR2-OVCAR8 cells were treated with a green fluorescent dye and the 4D12G1 mAb or an isotype control mAb. Apoptosis was assessed by live imaging using the IncuCyte S3 analyzer. 4D12G1 mAb induced substantial apoptosis at 16 hours (right panel) compared to isotype control mAb (left panel). (**B**) AMHR2-OVCAR8 cells were treated with different concentrations of the 4D12G1 mAb for 24 hours and Western blots of the cell lysates showed detection of the intact 116 kDa PARP-1 and its 89 kDa cleaved variant, consistent with apoptosis. Immunostaining with a β-actin antibody was used to confirm normalized lysate loading. (**C**) AMHR2-OVCAR8 cells were incubated with the 4D12G1 mAb for different time periods at either 37° C (left column) or 4° C (right column). Clustered patterns of cytoplasmic antibody-receptor complexes became increasingly more prominent at 2 and 3 hours after treatment at 37° C, but not at 4° C, and no staining occurred in cells treated with secondary antibody alone (right column, bottom panel). (**D**) AMHR2-OVCAR8 cells were incubated in either 10% normal human serum or 10% heat-inactivated human serum and treated for 4 hours with varying doses of either 4D12G1 mAb or isotype control mAb. Cell lysis mediated by CDC was measured by release of LDH activity and occurred only in cells treated with the 4D12G1 mAb. (**E**) AMHR2-OVCAR8 target cells were labeled with a green fluorescent dye and incubated with two different concentrations of 4D12G1 mAb or isotype control mAb. The cells were mixed with effector macrophages from C57BL/6 mouse bone marrow at an effector to target cell ratio of 10:1. Live target cells were analyzed by flow cytometry 3 days later for demonstrating ADCP. All error bars indicate ±SD. All experiments are representative of three experiments yielding similar data.

We previously reported that AMHR2-ED vaccination inhibited the growth of EOC tumors by antibody-mediated induction of programmed cell death [16]. To determine whether the 4D12G1 mAb acts similarly, we assessed real-time induction of apoptosis by live imaging of the AMHR2-OVCAR8 cells treated *in vitro* with the 4D12G1 mAb in DMEM supplemented with heat-inactivated serum. The results indicated that treatment for 16 hours with the 4D12G1 mAb induced a profound level of apoptosis in AMHR2-OVCAR8 cells (Figure 5A; right panel) compared to treatment for 16 hours with an isotype control IgG1 mAb (Figure 5A; left panel).

### Detection of PARP-1 cleavage

PARP-1 is a 116 kDa nuclear protein that serves as one of the main cleavage targets of caspase-3 resulting in generation of an 89 kDa fragment that can be detected as a signature of programmed cell death [22]. Thus, we examined whether the 4D12G1 mAb was able to cleave PARP-1 as a consequence of inducing an apoptotic signal. AMHR2-OVCAR8 cells were treated with different concentrations of the 4D12G1 mAb for 24 hours, and cell lysates were examined on Western blots for the presence of the cleaved 89 kDa PARP-1 fragment. The results showed that cleaved PARP-1 was detected in the lysates at 4D12G1 mAb doses as low as 1 μg/ml (Figure 5B). Immunostaining with a β-actin antibody (Sigma-Aldrich) was used to confirm normalized lysate loading.

### Internalization of AMHR2-mAb complexes

Since signaling through TGFβ superfamily receptors like AMHR2-ED involves endocytic receptor internalization [23], we examined whether this process occurs in the AMHR2-OVCAR8 cells treated with the 4D12G1 mAb. AMHR2-OVCAR8 cells were incubated with the 4D12G1 mAb for different periods of time at either 37° C (Figure 5C; left column) or 4° C (Figure 5C; right column). Complexes of the 4D12G1 mAb with the AMHR2 receptor were evident in the cytoplasm of the AMHR2-OVCAR8 cells by 1 hour after treatment with the 4D12G1 mAb at 37° C, and clustered patterns of cytoplasmic mAb-receptor complexes were increasingly more prominent 2 and 3 hours after treatment at 37° C. In contrast, treatment with the 4D12G1 mAb at 4° C showed virtually no cytoplasmic antibody-receptor complexes even 3 hours after treatment (Figure 5C; right column, middle panel). Staining was not observed in cells treated with secondary detection antibody alone (Figure 5C; right column, lower panel). Our results indicate that signaling induced by binding of the 4D12G1 mAb to AMHR2 results in apoptosis of EOC cells accompanied by endocytic internalization of the mAb-AMHR2 complexes and caspase-3 mediated cleavage of PARP-1.

### The 4D12G1 mAb induces EOC cell lysis by complement-dependent cytotoxicity (CDC)

The ability of the 4D12G1 mAb to mediate CDC was determined. AMHR2-OVCAR8 cells were treated *in vitro* for 4 hours with varying doses of the 4D12G1 mAb in the presence of either 10% normal human serum containing complement or 10% normal human serum heated to inactivate complement. Additional controls included AMHR2-OVCAR8 cells treated for 4 hours with varying doses of an isotype control mAb in the presence of either 10% normal human serum or 10% heat-inactivated human serum. Cell lysis was measured by release of lactate dehydrogenase activity. The results showed that the 4D12G1 mAb induced significantly increased cell lysis (*P* < 0.0001) in the presence of normal human serum containing complement compared to the lysis induced by the 4D12G1 mAb in the presence of heat-inactivated human serum (Figure 5D).

### The 4D12G1 mAb induces EOC cell lysis by antibody-mediated cellular phagocytosis (ADCP)

AMHR2-OVCAR8 target cells were labeled with a green fluorescent dye and incubated with two different concentrations of 4D12G1 mAb or isotype control mAb for 30 minutes in the absence of any serum. The washed cells were mixed with effector macrophages from C57BL/6 mouse bone marrow at an effector to target cell ratio of 10:1, and the percentage of live target cells was determined by flow cytometry 3 days later. The percentage of live target cells were significantly lower in AMHR2-OVCAR8 cells treated with the 4D12G1 mAb at 100 ng/ml (*P* = 0.001) and at 1000 ng/ml (*P* = 0.0002; Figure 5E) compared to the same treatments with the isotype control mAb. These data indicate that the 4D12G1 mAb is capable of inducing ADCP of EOC cells.

### The 4D12G1 mAb inhibits growth of xenografted human EOCs

**Figure 6:**
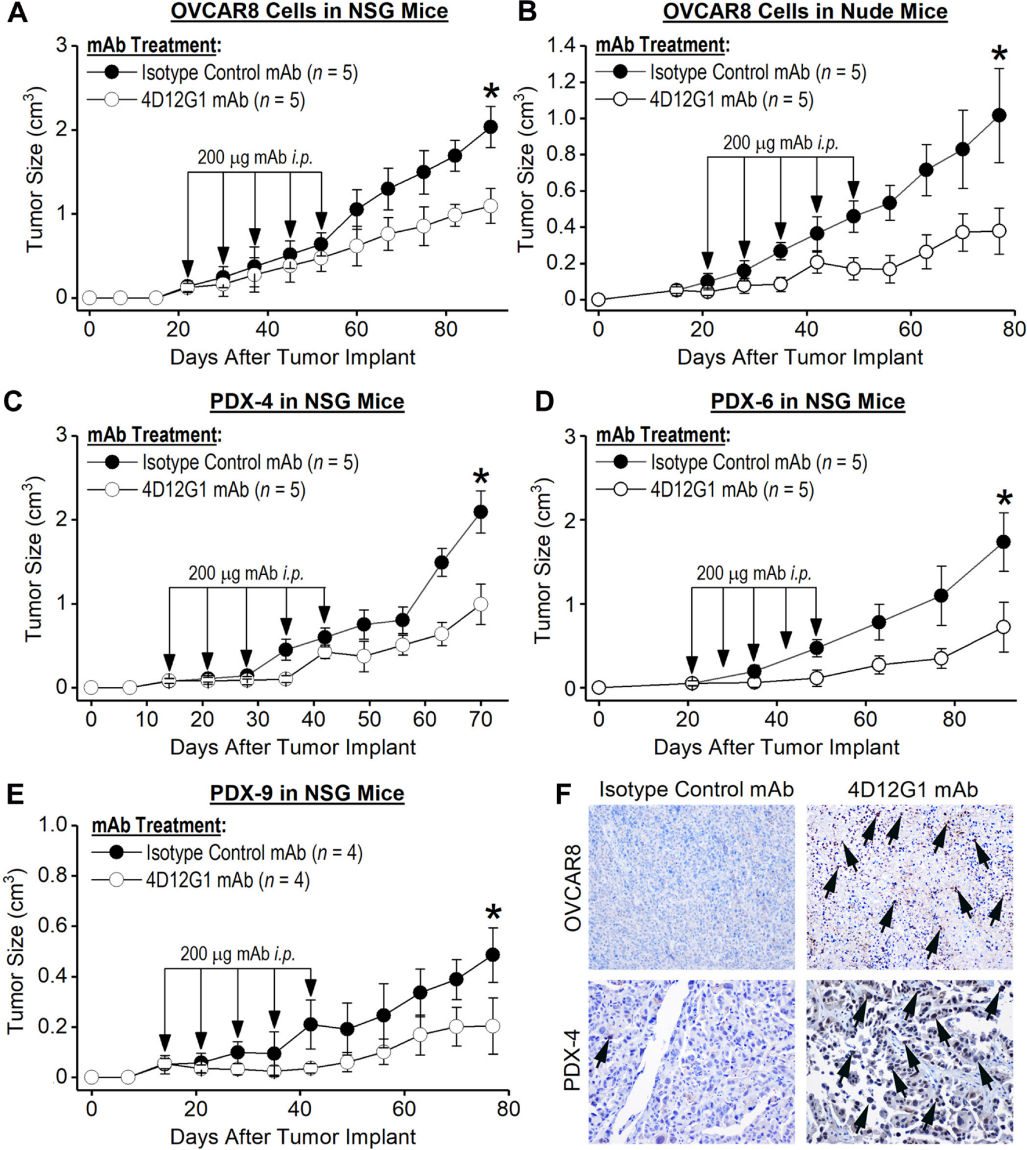
The 4D12G1 mAb inhibits growth of human EOC xenografts. Human EOC tumors were injected *s. c.* into immunodeficient mice. When tumors became palpable, mice were injected *i. p.* with 200 μg of either the 4D12G1 mAb or an isotype control mAb weekly for 5 continuous weeks. Treatment with the 4D12G1 mAb significantly inhibited the growth of OVCAR8 tumors in (**A**) severely immunodeficient NSG mice (*P* < 0.001) and in (**B**) T cell-deficient athymic nude mice (*P* < 0.0001). More importantly, treatment with the 4D12G1 mAb significantly inhibited the growth of three primary HGSOC tumors (*P* < 0.0001 in all cases) generated from recently diagnosed patients and xenografted into immunodeficient NSG mice including (**C**) PDX-4, (**D**) PDX-6, and (**E**) PDX-9. (**F**) Detection of caspase-3 positive cells in the OVCAR8 (upper row) and PDX-4 tumors (lower row) from NSG mice at 20× is shown by arrows in mice treated with the 4D12G1 mAb (right column) compared to mice treated with isotype control mAb (left column). Caspase-3 data shown are representative of three experiments yielding similar results. All error bars indicate ± SD.

OVCAR8 cells and human HGSOC tumors were injected *s. c.* into the flanks of immunodeficient mice. When tumors became palpable at about 50 mm^3^, mice were injected *i. p.* with 200 μg of either the 4D12G1 mAb or an isotype control mAb weekly for 5 continuous weeks. The results showed that treatment with the 4D12G1 mAb significantly inhibited the growth of OVCAR8 tumors in severely immunodeficient NSG mice (*P* < 0.001; Figure 6A) or T cell-deficient athymic nude mice (*P* < 0.0001; Figure 6B). It is interesting to note that the increased significant inhibition of tumor growth in athymic nude mice compared to NSG mice may reflect the absence of a hemolytic complement system, reduced dendritic cell function, and defective macrophage activity characteristic of NSG mice but not athymic nude mice thereby precluding contributions of CDC and ADCP immune mechanisms for anti-tumor response in the NSG mice [24]. Most importantly, treatment with the 4D12G1 mAb significantly inhibited the growth (*P* < 0.0001 in all cases) of three primary HGSOC tumors generated from recently diagnosed patients and xenografted into immunodeficient NSG mice including PDX-4 (Figure 6C), PDX-6 (Figure 6D), and PDX-9 (Figure 6E). Caspase-3 positive cells in an OVCAR8 tumor grown in an NSG mouse (Figure 6F, upper row) and in a PDX-4 tumor grown in an NSG mouse (Figure 6F, lower row) are shown by arrows (20 ×) in mice treated with the 4D12G1 mAb (Figure 6F, right column) compared to mice treated with an isotype control mAb (Figure 6F, left column). These caspase-3 data are representative of three experiments yielding similar results. All error bars indicate ± SD.

### Quantification of AMHR2 receptor density on the surface of EOC cells

It has been reported that as few as 4,000 AMHR2 receptors/cell are sufficient to mediate inhibition of EOC growth in xenografts by an AMHR2-ED-specific mAb [25]. To further examine this issue, we used the QIFIKIT^®^ series of coated beads (Agilent Technologies) and flow cytometry analysis to measure the cell surface density of AMHR2 receptors on EOC cells xenografted into immunodeficient mice. We found that cell surface densities of AMHR2 receptors were 2,674, 3,917, 22,286, 8,907, and 10,067 receptors/cell measured, respectively, on OVCAR8 cells xenografted into nude mice, OVCAR8 cells xenografted into NSG mice, and PDX-4, PDX-6 and PDX-9 HGSOC tumor cells xenografted into NSG mice. Thus, 2,674 AMHR2 receptors/cell are sufficient for the 4D12G1 mAb to mediate inhibition of EOC tumor growth in xenografts. More importantly, these data show that human HGSOC tumors have sufficient cell surface AMHR2 receptors to facilitate inhibition of tumor growth by the 4D12G1 mAb.

## DISCUSSION

Our study has characterized the immune features of the 4D12G1 IgG_1_ mAb including its specificity for AMHR2-ED, its ability to compete with the AMH cognate ligand for binding to AMHR2-ED, its ability to bind AMHR2-ED with a high affinity K_**D**_ of 119 pM, and its ability to function like the human AMH cognate ligand by inducing substantial apoptosis of AMHR2-expressing human EOC cells accompanied by endocytic internalization of receptor-mAb complexes and caspase-3 mediated cleavage of PARP-1. Most importantly, the 4D12G1 mAb significantly inhibits the growth of human EOC xenografts in immunodeficient mice through a predominant apoptotic mechanism, but it is also capable of inducing CDC and ADCP against EOC cells. Overall, these features qualify the 4D12G1 mAb as our prime candidate mAb for eventual use in human clinical trials for treating EOC.

Prior to use in clinical trials, the 4D12G1 mAb must undergo humanization by replacing the non-binding sequences of the mouse 4D12G1 mAb with the corresponding human sequences. Such rearrangements often cause conformational changes that impact the antigen-binding fragment F (ab′)_2_ resulting in decreased antigen binding affinity and modified antibody function. Such alterations occurred following humanization of the 12G4 mAb specific for the ^53^DRAQVEM^59^ sequence of AMHR2-ED [21, 25]. The 3C23K humanized version of the 12G4 mAb showed a loss of the ability to induce apoptosis of EOC cells but was still able to induce ADCC and ADCP [26, 27]. These mechanisms of antibody function rely heavily on the binding of the crystallizable fragment (Fc) region of the mAb to Fc receptors expressed on the surface of many cells of hematopoietic origin including macrophages and dendritic cells [28].

Despite loss of apoptotic signaling, the humanized 3C23K mAb was still capable of inducing a significant overall response rate in 15% of ovarian cancer PDXs [27]. This modest but significant overall response rate was due presumably to intact ADCC and ADCP activities resulting from glyco-engineering of the Fc binding domain with low fucosylation that increased Fc binding to Fc receptors, particularly the Fc gamma receptors for IgG (FcγR) [26, 27]. However, in a parallel study using the 3C23K mAb, no significant inhibition in the growth of EOC PDXs was evident [29]. The authors of this latter study proposed that efficacy of the 3C23K mAb in inhibiting tumor growth may be directly related to the density of AMHR2 receptors on the surface of EOC PDXs by showing that EOC cells expressing ≥ 4,000 AMHR2 receptors/cell surface provided a sufficient threshold density needed for inducing efficacy whereas cells expressing ≤ 2,300 receptors/cell surface failed to induce efficacy [29]. Our data indicate that threshold densities as low as 2,674 AMHR2 receptors/cell are sufficient for the 4D12G1 mAb to mediate inhibition of EOC growth in xenografts.

Despite the loss of function of the humanized 3C23K mAb (a. k. a. GM102) in different xenograft models, treatment with GM102 in a phase I clinical trial showed that 8/17 (47%) evaluable patients with ovarian cancer exhibited decreased tumor growth rates ranging from 45%–69% thereby indicating the potential for ovarian cancer immunotherapy with mAbs specific for AMHR2-ED [30] even if the humanized therapeutic mAb fails to induce apoptotic signaling [26, 27].

There are several differences between our 4D12G1 mAb and the original 12G4 mAb [21, 25] that was humanized and glycol-engineered to generate the 3C23K mAb (a. k. a. GM102). The binding domain for the 4D12G1 mAb recognizes the ^20^KTLGELL^26^ sequence of AMHR2-ED juxtaposed to the ^4^RRTCVFFEAPGV^15^ primary AMH binding site and adjacent to the ^34^RAIRCLYSR^42^ secondary AMH binding site (Figure 4B). In contrast, the 12G4 mAb and its humanized 3C23K variant recognizes the ^53^DRAQVEM^59^ sequence of AMHR2-ED located closer to the weaker tertiary and quaternary AMH binding sites ^46^GIWNL^50^ and ^83^PSPGSTLFT^91^, respectively, but far from the primary and secondary AMH binding sites (Figure 4B). Such distant recognition far from the strong AMH binding sites explains the inability of the 12G4 mAb to compete with AMH for receptor binding [26] and may help to explain the inconsistent induction of apoptosis by the 12G4 mAb and its humanized 3C23K variant [25, 29]. Thus, glycol engineering of the Fc binding region to increase binding affinity for Fc receptors appears to be a saving feature of the 3C23K mAb for inducing clinical efficacy by enhancing ADCP and NK-dependent ADCC functions [26]. The close proximity of the 4D12G1 binding site to the key primary and secondary AMH binding sites may provide a substantial advantage for inducing the consistently observed apoptotic signal created by the binding of the 4D12G1 mAb to the AMHR2-ED receptor.

The efficacy of humanized mAbs in treating human cancers is well established. Repeated administration of a single humanized mAb has proven to be effective in inhibiting the growth of numerous cancers [31]. The advantage of such therapeutic mAbs lies in the fact that they provide immediate immunity. Patients with established tumors can bypass a vaccination process that often takes weeks or months to achieve therapeutic antibody titers or may never produce clinically relevant antibody titers if the target antigen is not sufficiently immunogenic. Perhaps HER2 is the most prominent example of a target antigen that consistently fails to provide a clinically relevant immune response even after repeated vaccinations [32], but its mAbs, trastuzumab and pertuzumab, are effective against HER2+ tumors [33]. AMHR2-ED appears to have immunodominant features since a single vaccination induces extremely high polyclonal antibody titers and dramatically enhanced overall survival in both transplantable and autochthonous mouse models of EOC [16]. The EOC apoptosis induced by the 4D12G1 mAb certainly mimics but may never be as powerful as the apoptosis achieved by a polyclonal antibody response resulting from AMHR2-ED vaccination. Thus, one could consider a more optimized immunotherapeutic strategy against EOC that includes immediate induction of passive immunity by administration of the humanized 4D12G1 mAb followed by induction of active immunity by AMHR2-ED vaccination for providing delayed but permanent high titer antibodies sufficient to maintain long-term EOC growth inhibition. In any event, our studies show that the 4D12G1 mAb may provide an effective way to control the growth of human EOC.

We have shown that the 4D12G1 IgG_1_ mAb binds with antigen-specificity and high affinity to a sequence of human AMHR2-ED that lies adjacent to the primary and secondary binding sites recognized by the cognate AMH ligand. These binding features facilitate direct induction of apoptotic death of EOC cells by the 4D12G1 mAb and growth inhibition of primary human EOCs in PDXs in NSG mice. The 4D12G1 mAb also kills EOC cells through CDC and ADCP mechanisms not available in NSG mice. Our results indicate that the 4D12G1 mAb may serve in its humanized form as a novel, much needed, and effective immunotherapy against human EOC.

## MATERIALS AND METHODS

### Generation of recombinant human AMHR2-ED

The recombinant extracellular domain of human AMHR2 (rhAMHR2-ED; NCBI reference sequence: NG_015981.1; Uniprot Q16671) was generated and purified as an endotoxin-free C-terminal hexahistidine- (6xHis-) tagged fusion protein as previously described for the mouse variant [16].

### Immunization and generation of hybridomas

Female BALB/cJ mice were purchased from Jackson Laboratory (stock# 000651; Bar Harbor, ME) and immunized at 7–8 weeks of age with an emulsion of 100 μg rhAMHR2-ED in 100 μl USP grade water and 100 μl of Sigma Adjuvant System^®^ (#S6322; Sigma-Aldrich, St. Louis, MO). Each mouse received four identical immunizations performed at 2–3-week intervals with the first immunization administered subcutaneously (*s. c.*) and the three remaining booster immunizations administered intraperitoneally. Three days after the final immunization, spleen cells from immunized mice were fused with the mouse myeloma cell line, Sp2/O-Ag14 (#CRL-1581; ATCC, Manassas, VA) using polyethylene glycol (PEG; Sigma-Aldrich). The fused hybridoma cells were cultured in DMEM-20/HEPES/pyruvate medium supplemented with hypoxanthine-aminopterin-thymidine (HAT; Thermo Fisher, Waltham, MA) in humidified air at 37° C with 5% CO_2_. On day 15, when cells were ~25% confluent, hybridoma supernatants were tested by ELISA as described below for antigen-specific binding to rhAMHR2-ED using recombinant mouse β-casein as the specificity control as described previously [16]. Antigen-specific hybridomas were subsequently subcloned by limiting dilution, their supernatants were purified as described above, and the subcloned mAbs were isotyped using the Mouse Typer^®^ Isotyping Panel (Bio-Rad, Hercules, CA) as described previously [16]. Hybridoma clones were grown in DMEM with 10% fetal bovine serum (FBS) or ultra-low IgG FBS (HyClone, Logan, UT) until reaching log phase growth. The mAbs were purified from hybridoma supernatants by protein G chromatography (Genscript Biotech, Piscataway, NJ) using Pierce Protein G IgG Binding Buffer (Thermo Fisher) followed by elution using Pierce IgG Elution Buffer (Thermo Fisher). The mAbs were collected in 1M Tris-HCl at pH 9.0. All collected fractions were concentrated, reconstituted with saline at 1 mg/ml, passed through a 0.22 μm filter, and stored at −20° C until needed.

### Competitive ELISA

The specificity of the mAbs was determined by competitive ELISA. Briefly, mAbs were incubated with increasing concentrations of rhAMHR2-ED in liquid phase PBS containing 0.02% Tween-20 (Bio-Rad) for 24 hours at 4° C. The antigen-antibody mixtures were added to the 96-well Maxisorp microtiter plates (Corning Life Sciences, Tewksbury, MA) pre-coated with 4 μg/ml rhAMHR2-ED followed by blocking with 1% BSA (Sigma-Aldrich). Recombinant mouse β-casein was used as the specificity control [16]. Wells were then incubated with a secondary anti-mouse IgG conjugated with horseradish peroxidase (HRP; MilliporeSigma, Burlington, MA) followed by addition of 2,2′-Azino-bis (3-ethylbenzothiazoline-6-sulfonic acid) diammonium salt (ABTS) substrate (Sigma-Aldrich). Optical density was determined by absorbance at 405 nm.

### Human EOC cell lines

The human ovarian carcinoma 8 (OVCAR8) cell line was obtained commercially (ATCC #CVCL-1629) and authenticated by Genetica DNA Laboratories (Burlington, NC). OVCAR8 cells were grown in DMEM supplemented with 10% human sera from postmenopausal women (AMH low/free; BioIVT, Westbury, NY), 5% HEPES buffer (Sigma-Aldrich), 2 mM L-glutamine (Thermo Fisher), and 1% penicillin/streptomycin (Invitrogen, Carlsbad, CA). OVCAR8 cells were periodically tested for *Mycoplasma*, and their passage number did not exceed 10. The AMHR2-OVCAR8 cell line was generated by cloning transcript variant 1 of human AMHR2 (Accession #NM_020547; Origene, Rockville, MD) into pEZ-M68 vector (GeneCopoeia, Rockville, MD) and using this expression vector to transfect OVCAR8 cells using Lipofectamine™ 3000 (Thermo Fisher). AMHR2-OVCAR8 cells were grown as describe above for the parental OVCAR8 cells.

### Generation of EOC cell lysates and Western blotting

Lysates from cell lines, tissues, or from fresh human high grade serous ovarian carcinomas (HGSOCs) were prepared as previously described [34] by mincing and crushing in DMEM followed by treatment with collagenase (Sigma-Aldrich) and DNase (Invitrogen). The suspension was centrifuged to remove debris, the lysates were washed in PBS, and 25 μg of protein from each lysate was loaded into each lane of denaturing SDS-PAGE gels (Bio-Rad). Negative control lysates were generated from the human prostate cancer cell line C4-2 (ATTC^®^ #CRL-3314). Gels were transferred onto polyvinylidene difluoride (PVDF) membranes (Bio-Rad) and incubated with the 4D12G1 mAb at 0.001 μg/ml as primary antibody. In Figure 2D, a commercially available AMHR2-ED mAb (#sc-377413; Santa Cruz Biotechnology, Dallas, TX) at 1.0 μg/ml was used instead of the 4D12G1 mAb as the primary antibody for detecting AMHR2-ED. Since caspase-3-mediated cleavage of PARP-1 serves as a signature of programmed cell death [22], Western blots of lysates were used to detect caspase-3-mediated cleavage of PARP-1 in lysates of AMHR2-OVCAR8 cells that were treated with increasing concentrations of 4D12G1 mAb (1.0–10 μg/ml). A primary rabbit antibody was used for detecting the intact 116 kDa PARP-1 and its 89 kDa cleaved variant (Cell Signaling Technology, Danvers, MA). Secondary detection antibodies included treatment with a 1:10,000 dilution of an HRP-conjugated rabbit anti-mouse IgG (MilliporeSigma) for 4D12G1 and commercial AMHR2-ED primary antibodies or a 1:5,000 dilution of an HRP-conjugated goat anti-rabbit antibody (MilliporeSigma) for PARP-1 primary antibody. In all cases, lysate loading was normalized using a primary mAb against β-actin (Sigma-Aldrich), and blots were developed using the HyGLO™ chemiluminescence system (Thomas Scientific, Swedesboro, NJ).

### Immunohistochemistry

Immunostaining of human HGSOC and normal adjacent tissues was performed on formalin-fixed, paraffin-embedded, 5 μm sections as previously described [16]. Primary antibodies included 0.005 μg/ml of purified 4D12G1 mAb for visualizing AMHR2-ED distribution followed by detection with an HRP-conjugated anti-mouse IgG (Abcam, Cambridge, UK), and rabbit polyclonal caspase-3 antibody (R&D Systems, Minneapolis, MN) for detecting apoptotic cells followed by an HRP-conjugated anti-rabbit IgG (Abcam).

### Flow cytometry

Harvested cells were treated with Fc-receptor block (BD Biosciences, San Jose, CA) and incubated with either purified mAbs generated against rhAMHR2-ED or with isotype control mouse IgG. Cells were then treated with FITC-labeled goat anti-mouse IgG (BD Biosciences) and analyzed for antigen-specificity by flow cytometry using a FACSAria II flow cytometer and BDFacsDiva software (BD Biosciences). Positive control staining was performed using a commercially available mAb against AMHR2-ED (Abcam), whereas mouse IgG_1_ isotype antibodies (Thermo Fisher) with irrelevant specificities were used as negative controls. Recombinant human AMH (LSBio, Seattle, WA) and recombinant ovalbumin (Sigma-Aldrich) were used in competitive binding assays.

### Identification of the AMHR2-ED sequence recognized by the 4D12G1 mAb

Competitive ELISAs were used to determine the AMHR2-ED sequence recognized by the 4D12G1 mAb. A set of overlapping 16-mer peptides spanning the entire 132 amino acid sequence of human AMHR2-ED was generated. Each peptide shifted by one amino acid and all cysteine residues were replaced by serine to avoid disulfide bond formation (Thermo Fisher). Individual 16-mer peptides were plated in solid phase in microtiter wells, increasing concentrations of each 16-mer peptide were incubated with the 4D12G1 mAb in liquid phase, and ELISAs were performed as described above. The binding site of the 4D12G1 mAb was confirmed by competitive ELISA using overlapping peptides containing alanine substitutions for each amino acid spanning the immunoreactive region of AMHR2-ED. The substituted peptides were used in liquid phase with the 4D12G1 mAb to determine binding inhibition to rhAMHR2-ED in solid phase. The AMHR2-ED binding sequence of the 4D12G1 mAb and the critical AMHR2-ED residues for such binding were confirmed by SPOT peptide arrays [18]. Peptides spanning the AMHR2-ED 9–40 immunoreactive region and ranging from 4-16-mers in length were synthesized > 90% pure on cellulose membranes (JPT Peptide Technologies, Berlin, Germany), treated overnight with blocking buffer (Thermo Fisher), followed by the treatment with the 4D12G1 mAb at 1.0 μg/ml for 3 hours at room temperature, followed by three washes with PBS containing 0.01% Tween-20 (Bio-Rad). The membranes were then treated with a 1:10,000 dilution of an HRP-conjugated rabbit anti-mouse IgG (MilliporeSigma) followed by three washes with PBS containing 0.01% Tween-20 (Bio-Rad). Bound antibody was detected using the HyGLO chemiluminescence system (Thomas Scientific).

### Surface plasmon resonance (SPR)

The equilibrium dissociation constant (K_**D**_) between the 4D12G1 mAb and rhAMHR2-ED was determined by high sensitivity binding in real-time by SPR measured at 25° C using a Biacore 3000 instrument (GE Healthcare Bio-Sciences, Piscataway, NJ). Briefly, the 4D12G1 mAb at 300 nM was diluted in sodium acetate at pH 5.0 and covalently immobilized on carboxymethylated dextran over a gold surface using an aqueous solution of *N*-ethyl-*N*′-(3-dimethylaminopropyl) carbodiimide (EDC) and *N*-hydroxysuccinimide (NHS; Sigma-Aldrich). The rhAMHR2-ED receptor analyte at different concentrations (10^−1^−10^3^ nM) in a running buffer containing 10 mM HEPES at pH 7.4, 150 mM NaCl, 3 mM EDTA, and 0.005% P20 detergent was captured with a 30 ml/minute flow over the immobilized 4D12G1 ligand. Regeneration of flow cells was performed using 10 mM glycine (Sigma-Aldrich) at pH 1.7. All sensorgrams were corrected by subtracting the low signal of the control flow cell and the dissociation curve of the 4D12G1 mAb. The K_**D**_ values, taking into account affinity and avidity, were calculated using a Langmuir 1:1 fitting model and BIAevaluation 3.2 software (GE Healthcare Bio-Sciences).

### Molecular modeling of human AMHR2-ED

Molecular modeling of AMHR2-ED was performed using Phyre2 [19] and 3DLigandSite [20] tools. Four templates were chosen for modeling AMHR2-ED based on heuristics to maximize confidence, percentage identity, and alignment coverage. These templates included: 1) 1bte for the extracellular domain of the activin type 2 receptor (ACVR2); 2) 4fao for activin receptor-like kinase 1 (ALK1); 3) 2hlq for bone morphogenetic protein receptor type 2 (BMPR2); and 4) 2h62 for the ternary ligand-receptor complex of bone morphogenetic protein 2 (BMP2). The structure modeling with 100% confidence was chosen for subsequent analysis. The AMHR2-ED protein fragment was visualized using the PyMOL Molecular Graphics System, version 1.5.0.5 [20].

### Real-time imaging of programmed cell death

AMHR2-OVCAR8 cells were incubated with the green fluorescent dye IncuCyte Cytotox (Essen BioScience, Ann Arbor, MI) for 16 hours in complement-free DMEM at 37° C with 10 μg/ml of either the 4D12G1 mAb or an IgG_1_ isotype control mAb. Cell death was imaged in real-time using the IncuCyte S3 live-cell analysis.

### Internalization of the 4D12G1 mAb

Internalization of the 4D12G1 mAb following binding to AMHR2-ED in AMHR2-OVCAR8 cells was examined by immunofluorescence. AMHR2-OVCAR8 cells in log-phase growth were treated with 4D12G1 mAb at 5 μg/ml in PBS containing 0.1% BSA (Sigma-Aldrich) for different times at either 37° C or 4° C. After several washes, cells were fixed with 3.7% formaldehyde and permeabilized with PBS containing 0.4% Triton-X100 (Sigma-Aldrich). After washing, cells were incubated with an Alexa Fluor 488-conjugated anti-mouse IgG (Abcam) for 1 hour at room temperature. Nuclei were stained with 4′,6-diamidino-2-phenylindole (DAPI; Sigma-Aldrich) for 30 minutes at room temperature, coverslips were mounted on slides using Vectashield Antifade Mounting Media (Vector Laboratories, Burlingame, CA), and the cells were visualized by fluorescent microscopy.

### Complement-dependent cytotoxicity (CDC)

AMHR2-OVCAR8 cells were washed and treated with different concentrations of the 4D12G1 mAb or an isotype control mAb. The cells were then incubated in 96-well flat-bottom plates (Corning Life Sciences) with either 10% normal human sera or 10% heat-inactivated normal human sera. After 4 hours, supernatant samples were removed and the cultures were treated with lysis buffer. Extracellular and total lactate dehydrogenase (LDH) activities were quantified using the CyQUANT LDH Cytotoxicity Assay kit (Thermo Fisher) that measures LDH catalyzed conversion of lactate to pyruvate and subsequent reduction of a tetrazolium salt to a red formazan product measured at 490 nm. The level of formazan formation is directly proportional to the amount of LDH activity released into the medium and the percent of LDH released was determined by the measured total LDH activity detected in the cultured cells.

### Antibody-dependent cellular phagocytosis (ADCP)

Target AMHR2-OVCAR8 cells were labeled with CellTracker Green CMFDA Dye (Thermo Fisher) and incubated with varying concentrations of the 4D12G1 mAb or an isotype control mAb in DMEM supplemented with 10% FBS. After 30 minutes, differentiated macrophages derived from C57BL/6 bone marrow (Sciencell Research Laboratories, Carlsbad, CA) were added as effector cells at an effector to target cell ration of 10:1. After 3 days, ADCP was determined by flow cytometry determination of the percentage of live target cells.

### OVCAR8 and patient-derived xenografts (PDXs)

Female athymic nude mice (NU/J, JAX stock #002019) were purchased at 6–8 weeks of age (Jackson Laboratory) for use as recipients of OVCAR8 xenografts. Female NOD-scid IL2Rgamma^null^ (NSG) mice were purchased at 6–8 weeks of age from the Cleveland Clinic Biological Resources Unit (Cleveland, OH). 5 × 10^6^ OVCAR8 cells were injected *s. c.* into the flank of (A) NSG mice and (B) athymic nude mice aged 6–8 weeks. When tumors became palpable at about 50 mm^3^, mice were injected intraperitoneally (*i. p.*) with 200 μg of 4D12G1 mAb or isotype control mAb once a week for 5 continuous weeks. Experiments were terminated when the tumor reached 2000 mm^3^. PDXs were generated by implanting HGSOCs from patients into female NSG mice aged 6–8 weeks. Freshly excised tumors were cut into approximately 2 mm^3^ sized fragments and implanted *s. c.* into the flank area followed by a drop of Matrigel chilled at 4° C (Corning Life Sciences). Mice implanted directly with patient tumor samples were designated as P1. Tumor growth was accessed periodically using a Vernier caliper. P1 endpoint tumors were dissected and implanted into new female NSG mice as described above. Passages ≥ P2 were used to study the efficacy of *i. p.* injection of 200 μg of the 4D12G1 mAb or an isotype control mAb once a week for 5 continuous weeks starting when the tumor became palpable. All human tissues were obtained with prior approval from the Cleveland Clinic’s Internal Review Board (IRB #12–1404) and were considered exempt research involving use of existing data without recording subject identifiers. All protocols for animal research met with the prior approval of the Cleveland Clinic’s Institutional Animal Care and Use Committee of the Cleveland Clinic (IACUC) in compliance with the Public Health Service policy on humane care and use of research animals (IACUC #2018–2090).

### Quantification of AMHR2 receptor density on the surface of EOC cells

AMHR2 receptor density was measured on tumor cells obtained from OVCAR8 xenografts and PDXs using the QIFIKIT^®^ series of 6 bead populations coated with different but well-defined quantities of a mouse mAb (Agilent Technologies, Carpinteria, CA). The specimen cells were labelled at a saturating concentration with the 4D12G1 mAb as the primary antibody so that the number of bound 4D12G1 mAb molecules corresponded to the number of antigenic sites. The cells were then incubated in parallel with the QIFIKIT^®^ beads, with a FITC-conjugated polyclonal goat anti-mouse immunoglobulin, and with a goat F (ab′)_2_ at saturating concentration. A calibration curve was constructed by plotting the mean fluorescence intensity (MFI) of the individual bead populations against the number of mAb molecules on the beads. The number of antigenic sites on the specimen cells were then determined by interpolation and expressed as the number of AMHR2 receptors/cell.

### Biostatistical analysis

Differences in tumor sizes, CDC assays, and competitive ELISA analysis of epitope mapping were compared using two-way analysis of variance (ANOVA). Differences in ADCP assays and binding of monoclonal antibodies to human cells by flow cytometry were compared using the one sample student *t*-test. All differences were considered statistically significant when *P* ≤ 0.05 (95% confidence interval). All experiments were repeated 3 times independently.

## Abbreviations

6xHis: hexahistidine
ABTS: 2,2′-Azino-bis(3-ethylbenzothiazoline-6-sulfonic acid) diammonium salt
ACVR2: activin type 2 receptor 2
ADCP: antibody-dependent cellular phagocytosis
ALK1: activin receptor-like kinase 1
AMH: anti-Müllerian hormone
AMHR2: anti-Müllerian hormone receptor type II
AMHR2-CD: cytoplasmic domain of anti-Müllerian hormone receptor type II
AMHR2-ED: extracellular domain of anti-Müllerian hormone receptor II
ANOVA: analysis of variance
ATCC: American Type Culture Collection
BMP2: bone morphogenetic protein 2
BMPR2: bone morphogenetic protein receptor type 2
C4-2: a human prostate cancer cell line
CDC: complement-dependent cytotoxicity
DAPI: 4′,6-diamidino-2-phenylindole
EDC: *N*-ethyl-*N*′-(3-dimethylaminopropyl)carbodiimide
F(ab′)_2_: antigen-binding fragment of IgG
FBS: fetal bovine serum
Fc: crystallizable fragment of IgG
FcγR: Fc gamma receptors for IgG
HAT: hypoxanthine-aminopterin-thymidine medium
HGSOC: high grade serous ovarian carcinoma
HRP: horseradish peroxidase
IACUC: institutional animal care and use committee
*i.p.*: intraperitoneally
IRB: internal review board
K_**D**_: equilibrium dissociation constant
LDH: lactate dehydrogenase
mAb: monoclonal antibody
MFI: mean fluorescence intensity
MISIIR: Müllerian inhibitory substance type II receptor
NHS: *N*-hydroxysuccinimide
NSG: NOD-scid IL2Rgamma^null^ immunodeficient mouse
OVCAR8: human ovarian carcinoma 8 EOC cell line
PARP-1: poly (ADP-ribose) polymerase-1
PDX: patient-derived xenografts
PEG: polyethylene glycol
rh: recombinant human
*s.c.*: subcutaneously
SPR: surface plasmon resonance
TGFβ: transforming growth factor-beta
