# Correction: Immunotherapy of ovarian cancer with a monoclonal antibody specific for the extracellular domain of anti-Müllerian hormone receptor II

**DOI:** 10.18632/oncotarget.28246

**Published:** 2022-07-28

**Authors:** Suparna Mazumder, Valerie Swank, Anton A. Komar, Justin M. Johnson, Vincent K. Tuohy

**Affiliations:** ^1^Department of Inflammation and Immunity, Lerner Research Institute, Cleveland Clinic, Cleveland, OH, USA; ^2^Department of Molecular Medicine, Cleveland Clinic Lerner College of Medicine of Case Western Reserve University, Cleveland, OH, USA; ^3^Case Comprehensive Cancer Center, Cleveland, OH, USA; ^4^Department of Biological, Geological and Environmental Sciences, Cleveland State University, Cleveland, OH, USA; ^5^Center for Gene Regulation in Health and Disease, Cleveland State University, Cleveland, OH, USA

**This article has been corrected:** In Figure 2C, one field of the 1-Normal adjacent tissue section image (row 1, right panel) was accidentally incorporated into the 3-Normal adjacent tissue section stained image (row 3, right panel). The corrected Figure 2, produced from the original data, is shown below. The authors declare that these corrections do not change the results or conclusions of this paper.

Original article: Oncotarget. 2020; 11:1894–1910. 1894-1910. https://doi.org/10.18632/oncotarget.27585

**Figure 2:**
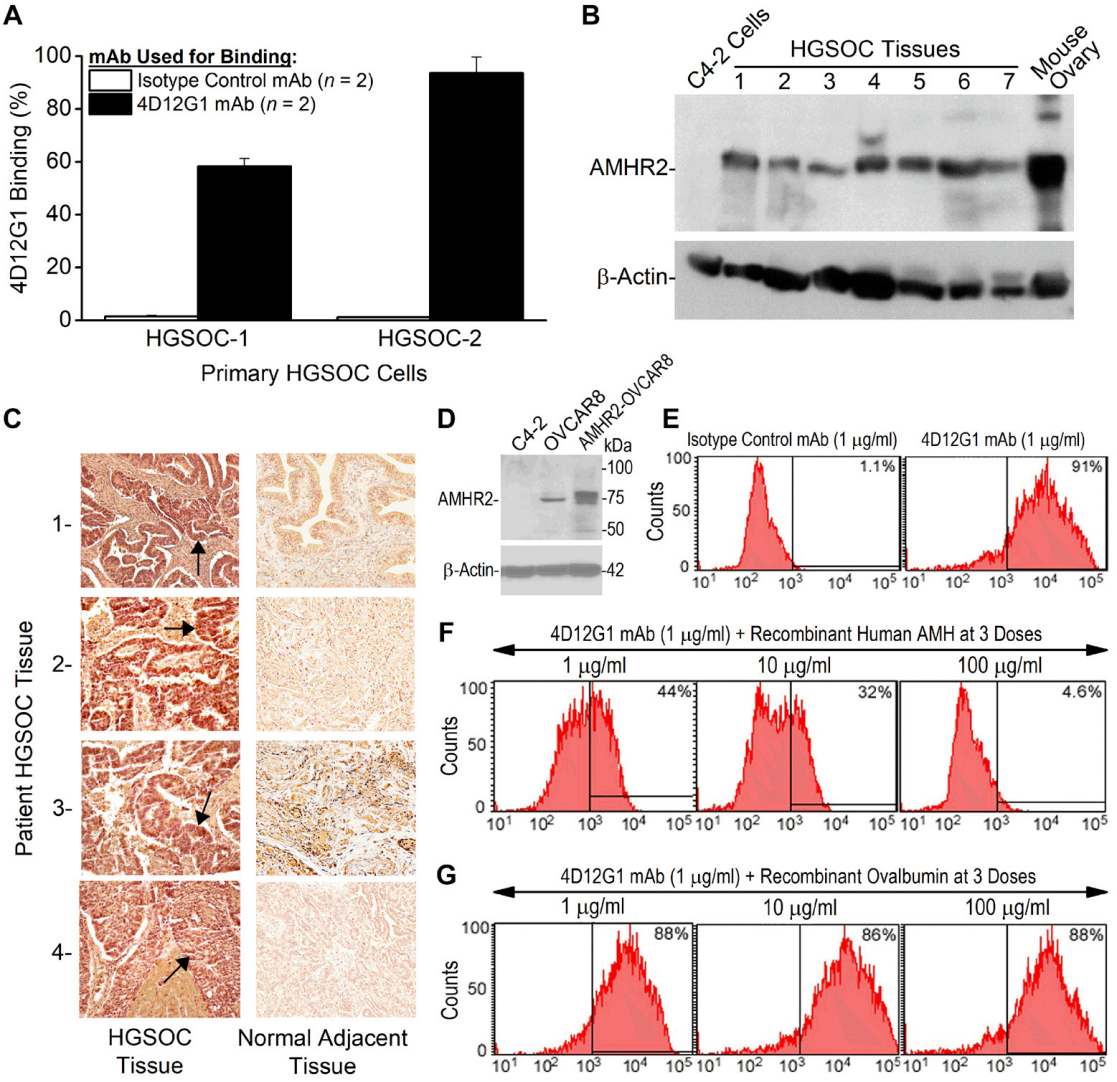
The 4D12G1 mAb recognizes AMHR2-ED in human EOC and competes with AMH for b inding to AMHR2-ED. (**A**) Flow cytometry analysis showing that the 4D12G1 mAb binds to the majority of cells generated from two primary HGSOC tissues examined. Error bars indicate ± SD. (**B**) The 4D12G1 mAb was used in Western blots of seven different HGSOC tissue lysates (25 μg protein/lane) with a positive control lysate generated from a young C57BL/6 ovary and a negative control lysate generated from C4-2 human prostate cancer cells. Immunostaining with a β-actin antibody was used to confirm normalized lysate loading. The Western blots shown are representative of three experiments that provided similar results. (**C**) The 4D12G1 mAb was used in immunohistochemical staining (20×) of tissue sections from four HGSOC patients (left column) and their normal adjacent fallopian tube tissues (right column). Arrows indicate staining of the tumor parenchyma. The stromal areas of the EOC tumors were not immunostained nor were all areas of the normal adjacent fallopian tube tissues. All experiments were performed three times yielding similar results. (**D**) Western blot analysis of lysates from OVCAR8 cells and AMHR2-OVCAR8 cells with lysates from C4-2 prostate cancer cells used as controls and immunostaining with a β-actin antibody was used to confirm normalized lysate loading. Flow cytometry analysis showed that: (**E**) the 4D12G1 mAb binds to 91% of AMHR2-OVCAR8 cells; (**F**) the AMH cognate ligand for AMHR2-ED effectively competes in a dose-dependent manner with the 4D12G1 mAb for binding to AMHR2-OVCAR8 cells; and (**G**) recombinant ovalbumin failed to compete with the 4D12G1 mAb for binding to AMHR2-OVCAR8 cells. Data are representative of three independent experiments yielding similar results.

